# Genome Sequences of Two Azospirillum sp. Strains, TSA2S and TSH100, Plant Growth-Promoting Rhizobacteria with N_2_O Mitigation Abilities

**DOI:** 10.1128/MRA.00459-19

**Published:** 2019-08-08

**Authors:** Nan Gao, Weishou Shen, Tomoyasu Nishizawa, Kazuo Isobe, Yong Guo, Hanjie Ying, Keishi Senoo

**Affiliations:** a National Engineering Research Center for Biotechnology, School of Biological and Pharmaceutical Engineering, Nanjing Tech University, Nanjing, China; b Jiangsu Key Laboratory of Atmospheric Environment Monitoring and Pollution Control, Collaborative Innovation Center of Atmospheric Environment and Equipment Technology, School of Environmental Science and Engineering, Nanjing University of Information Science and Technology, Nanjing, China; c Department of Applied Biological Chemistry, Graduate School of Agricultural and Life Sciences, The University of Tokyo, Tokyo, Japan; d Department of Bioresource Science, College of Agriculture, Ibaraki University, Ibaraki, Japan; e Collaborative Research Institute for Innovative Microbiology, The University of Tokyo, Tokyo, Japan; University of Southern California

## Abstract

Azospirillum sp. strains TSA2S and TSH100 are plant growth-promoting rhizobacteria with the capacity to mitigate N_2_O from agricultural soil. They were isolated from the rhizosphere of paddy soil in Tokyo, Japan. Here, we present the genome sequences of these two strains.

## ANNOUNCEMENT

Plant growth-promoting rhizobacteria (PGPR) are a group of rhizosphere bacteria which can improve plant growth, suppress invading pathogens, and improve plant abiotic stress tolerance and productivity (1–4). Studies have shown that the inoculation of soil with PGPR strains with nitrous oxide (N_2_O)-reducing ability decreases N_2_O emissions (2, 5, 6). As PGPR, Azospirillum sp. strains TSA2S and TSH100, originally isolated from the rhizosphere of paddy soil in Tokyo, Japan (7), possess the ability to improve plant growth and mitigate N_2_O from soil (2, 6). Thus, these two strains could be explored as environmentally friendly biofertilizers. Here, we present the genome sequences of TSA2S and TSH100.

A single colony of each strain was grown in 5 ml nutrient broth with NaNO_3_ and sodium succinate culture medium (peptone 5 g liter^−1^ and beef extract 3 g liter^−1^ containing 0.3 mM NaNO_3_ and 4 mM sodium succinate, pH 7.0) at 26°C and 220 rpm. Twenty-four to 48-hour cultures of the 2 strains were collected. The genomic DNA was extracted with a DNeasy blood and tissue kit (Qiagen, Germany) according to the manufacturer’s protocol. A SMRTbell library of 20-kb insert size was constructed with the template prep kit v1.0 and the BluePippin size selection system using standard protocols. The genomes were sequenced at Macrogen Japan with a PacBio RS II DNA sequencing system using C4 chemistry. In order to use only the PacBio long reads, FALCON software (v0.2.1) (8), which is a *de novo* genome assembler, was applied with default parameters except that daligner selected overlap detection and error correction of the raw reads. When the ends of each contig are overlapped, the contigs are connected to form a circular DNA molecule. When there is no sign of overlapping, the contig might have been originally linear, or there might be gaps at the end of the contig. The genome sequences were annotated using the NCBI Prokaryotic Genome Annotation Pipeline (PGAP, v4.8) with the best-placed reference protein set (GeneMarkS-2+) (9, 10).

Strain TSA2S, with about 171-fold genome coverage, had a circular chromosome of 2,804,606 bp, 6 chromids (11, 12), and 3 plasmids. A total of 7,349 protein-coding sequences (CDSs), 82 tRNA genes, and 29 rRNA genes were discovered. Strain TSH100, of about 192-fold genome coverage, had a circular chromosome of 2,712,114 bp, 5 chromids (11, 12), and 2 plasmids. A total of 6,221 CDSs, 80 tRNA genes, and 26 rRNA genes were discovered. The genomes of *Azospirillum* spp. constitute multiple replicons; the largest replicon has all the features of a bacterial chromosome, whereas the chromid definition applies to the corresponding replicon, i.e., plasmid-type maintenance replication systems, the presence of essential genes, and a nucleotide composition close to that of the chromosome (11, 12). The complete denitrification gene sets were identified on the chromosomes of TSA2S and TSH100. The whole genomes contain gene clusters encoding nitrogen fixation, a two-component system relative to quorum sensing, bacterial chemotaxis, and genes encoding lipopolysaccharide biosynthesis that may be involved in plant-microbe communications for symbiosis (13). The whole genomes contain genes encoding carbon fixation. The whole-genome sequences are of critical importance for revealing the molecular mechanisms of TSA2S and TSH100 for the promotion of plant growth and the mitigation of N_2_O emissions from agricultural soil.

### Data availability.

The whole-genome sequences of strains TSA2S and TSH100 have been deposited in GenBank under the accession numbers listed in Table 1. The raw reads have been registered and submitted to the Sequence Read Archive (SRA) under the accession numbers listed in Table 1.

**TABLE 1:** Genome features and GenBank accession numbers of two *Azospirillum* sp. strains, TAS2S and TSH100

Strain	Contig name	*N*_50_	No. of reads	Length (bp)	GC content (%)	Circular contig	Alias	No. of CDSs	No. of tRNAs	No. of rRNAs	GenBank accession no.	SRA accession no.
*Azospirillum* sp. TSA2S		11,698	160,553	8,102,478			Genome	7,349	82	29		SRR8886125
	Contig 1			2,804,606	67.5	Yes	Chromosome	2,617	46	6	CP039650	
	Contig 2			927,212	66.2	Yes	Chromid 1^*a*^	844	3	3	CP039647	
	Contig 3			903,871	67.4	Yes	Chromid 2^*a*^	776	9	5	CP039649	
	Contig 4			855,324	67.5	Yes	Chromid 3^*a*^	696	8	9	CP039648	
	Contig 5			682,730	68.2	No	Chromid 4^*a*^	662	2	3	CP039645	
	Contig 6			583,836	68.1	No	Chromid 5^*a*^	530	6	0	CP039642	
	Contig 7			533,779	67.4	Yes	Chromid 6^*a*^	497	8	3	CP039646	
	Contig 8			353,832	63.7	No	Plasmid 1	361	0	0	CP039651	
	Contig 9			351,631	66.8	Yes	Plasmid 2	268	0	0	CP039644	
	Contig 10			105,657	63.7	No	Plasmid 3	98	0	0	CP039643	
*Azospirillum* sp. TSH100		10,960	170,174	7,166,382				6,221	80	26		SRR8886132
	Contig 1			2,712,114	67.2	Yes	Chromosome	2,508	46	6	CP039634	
	Contig 2			1,162,302	67.2	No	Chromid 1^*a*^	978	11	8	CP039635	
	Contig 3			917,252	66.8	Yes	Chromid 2^*a*^	745	6	6	CP039636	
	Contig 4			912,147	67.7	Yes	Chromid 3^*a*^	760	3	3	CP039637	
	Contig 5			583,445	68.5	Yes	Chromid 4^*a*^	524	6	0	CP039638	
	Contig 6			498,809	67.7	No	Chromid 5^*a*^	412	8	3	CP039639	
	Contig 7			325,924	67.5	No	Plasmid 1	226	0	0	CP039640	
	Contig 8			54,389	68.2	No	Plasmid 2	68	0	0	CP039641	

a The GenBank sequences for *Azospirillum* sp. TSA2S, chromids 1 through 6, and those for strain TSH100, chromids 1 through 5, are called chromosomes and noted as chromids because NCBI does not have a chromid qualifier.
